# Comparison of enteral nutrition with combined enteral and parenteral nutrition in post-pancreaticoduodenectomy patients: a pilot study

**DOI:** 10.1186/1475-2891-8-24

**Published:** 2009-06-11

**Authors:** Shigeyuki Nagata, Kengo Fukuzawa, Yukio Iwashita, Akira Kabashima, Tadahiko Kinoshita, Kenzo Wakasugi, Yoshihiko Maehara

**Affiliations:** 1 Department of Surgery and Science, Graduate School of Medical Sciences, Kyushu University, Fukuoka, Japan; 2 Department of Surgery, Oita Red Cross Hospital, Oita, Japan

## Abstract

**Background:**

Many clinical studies have demonstrated that early postoperative enteral nutrition (EN) improved the postroperative course. Post-pancreaticoduodenectomy (PD), patients tend to suffer from postoperative nausea, abdominal distention, and diarrhoea, causing difficulty in the introduction of EN. In this pilot study, we investigated the appropriate nutritional mode post-pancreatic surgery.

**Methods:**

Between October 2006 and March 2007 2 postoperative nutritional methods were implemented in 17 patients in a prospective single-centere study. Eight patients received only enteral nutrition (EN group) and 9 patients received enteral nutrition combined with parenteral nutrition (EN + PN group).

**Results:**

There were no differences in the patient characteristics and postoperative morbidity between the 2 groups. The rate of discontinuance of enteral feeding was significantly high in the EN group, and the duration of enteral feeding was significantly longer in the EN + PN group. The central venous line was retained for a significantly longer period in the EN + PN group, but there was no difference in the frequency of catheter-related infection between the 2 groups.

**Conclusion:**

EN combined with PN is more adequate for patients after pancreatic surgery.

## Background

In current digestive surgical practice, the benefits of enteral nutritional support, in comparison with parenteral nutrition, are widely recognized. Recent experiences have shown that early postoperative enteral nutrition (EN) enhanced immunocompetence, reduced clinical infection rates, and maintained gut structure and function, and it can potentially attenuate catabolic stress responses in patients after surgery [1-5]. Although many studies have reported that catheter-associated infective complications are more frequently elicited by total parenteral nutrition (TPN), some studies have reported that the TPN-associated infections can be attributed to hyperglycemia and caloric overload, and that insulin therapy can alleviate these infections [6,7]. In addition, enteral nutrition is believed be safer and less expensive than parenteral nutrition. However, postoperative total enteral feeding is associated with complications such as diarrhoea, abdominal distention, and abdominal cramps. On the basis of our experience and the findings of previous studies [8,9], we believed that these symptoms worsened with increasing caloric intake and finally lead to discontinuance of enteral feeding.

Pancreaticoduodenectomy (PD) is associated with a high incidence of postoperative complications, even when the procedure is performed at high-volume centers. An overall morbidity rate of 48% can be anticipated at major centers, while the mortality rate in these centers is less than 4%. The high rate of complications can delay postoperative resumption of adequate oral food intake. Moreover, cancer or chronic pancreatitis patients who are candidates for PD often have associated comorbidities such as diabetes, jaundice, and protein-energy malnutrition [9,10]. Taken together, these issues present the case for artificial nutritional support. However, there is very limited clinical data on postoperative feeding after major pancreatic resections [8-10]. Therefore, we believe that the optimal nutritional method after pancreatic surgery has still not been identified.

In our institution, which is a high-volume center for pancreatic surgery, the patients who underwent PD, including pylorus-preserved PD (PpPD), routinely received enteral feeding from the early postsurgical period. However, there was no clinical regimen for enteral nutrition, and the menu for enteral feeding, which was prescribed by the doctors, was unique for each patient. We retrospectively examined 30 patients who underwent PD and PpPD in the 18 months prior to this study. It was observed that enteral feeding was discontinued and changed to TPN in many of these patients because of diarrhoea and abdominal distention.

In this prospective pilot study, we aimed to identify the ideal post-PD nutritional mode that could be administered without any interruptions and we compared the clinical outcomes, nutritional status, and immunological status of the 2 modes of postoperative nutrition, namely, enteral nutrition and enteral nutrition combined with parenteral nutrition.

## Methods

### Patients

We prospectively investigated 17 patients (12 men and 5 women; mean age, 68.3 years; range, 43–86 years) who had undergone PD or PpPD for peri-ampullary tumors between October 2006 and March 2007 at the Oita Red Cross Hospital. Among these 17 patients, there were 10 cases of pancreatic invasive ductal carcinoma, 5 cases of cholangiocarcinoma, and 2 cases of chronic pancreatitis with inflammatory mass (Table 1). The exclusion criteria included clinically relevant organ failure, ongoing infections, and inflammatory bowel diseases. Fully informed consent was obtained from all the patients. After surgery, randomization was performed using sealed envelopes. The patients were divided into 2 groups: those who received only enteral nutrition (EN group, n = 8) and those who received both enteral and parenteral nutrition (EN + PN group, n = 9).

**Table 1:** Postoperative complications

**Complication**	**EN group** **(n = 8)**	**EN+PN group** **(n = 9)**
Surgery related complications		
Pancreatic leakage (minor leakage)	0	1
Anastomotic leakage (minor leakage)	1	0
Ileus	1	0
Ulcer at anastomotic portion	0	1
Wound infection	3	1
General complications		
Pulmonary	1	0
Total number of patients with complications	5	4
Mortality	1	0
		
No significant differences noted.		

### Surgical procedure

The standard PD consisted of distal gastrectomy encompassing the duodenum and common bile duct, the gallbladder, and the head, neck, and the uncinate process of the pancreas; lymphadenectomy was also performed. Standard lymph-node dissection was performed according to the definition provided by Pedrazzoli et al. [11]. In PpPD, the duodenum was divided at a point 2 cm away from the pylorus. The passage was reconstructed by pancreatogastrostomy, end-to-side hepaticojejunostomy, end-to-end gastrojejunostomy in PD or pylorojejunostomy in PpPD, and an end-to-side jejunojejunostomy using the Roux-en-Y-technique (30 cm aborally from the gastrojejunal anastomosis). For postoperative nutritional support, all the patients received needle-catheter jejunostomy at the end of the operation Then, 8-Fr silicone jejunal tubes were inserted from the proximal portion of the jejunojejunostomy and fixed by the modified Witzel technique. The opposite tip was extracorporeally induced via an abdominal wall at the left flank.

### Postoperative nutrition

**Figure 1:**
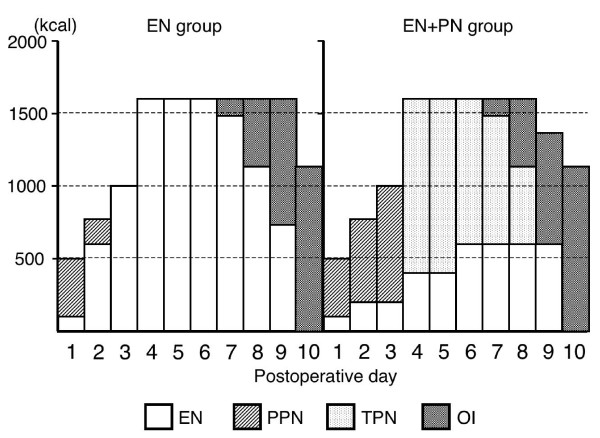
**Schedules of postoperative nutrition**. The infusion of 500 ml of 5% liquid glucose commenced within 12 h of surgery and enteral feeding was started on POD 2 in both groups. All patients in both groups reached the maximum volume of total caloric intake (derived using Harris-Benedict equation), on POD 4. In the EN group, the total volume of EN was 600 kcal/day on POD 2 and 1000 kcal/day on POD 3 with PPN. In the EN + PN group, EN started at 200 kcal/day and was increased every 2 days to a maximum volume of 600 kcal/day from POD 6. The patients received PPN until POD 3 or TPN from POD 4 to compensate for the caloric shortage. Oral intake started on POD 7. TPN was stopped when oral intake was over 500 kcal/day and enteral nutrition was stopped when oral intake was over 1000 kcal/day. EN: enteral nutrition, PPN: peripheral parenteral nutrition, TPN: total parenteral nutrition, OI: oral intake.

All the patients in both the groups received enteral feeding with/without parenteral nutrition as per the schedule outlined in Fig. 1. Briefly, the infusion of 500 ml of 5% glucose commenced within 12 h after surgery, and enteral feeding was started on postoperative day (POD) 2 in both the groups. All the patients in the 2 groups reached the maximum value of total caloric intake (obtained by Harris-Benedict equation) on POD 4. In the EN group, total caloric intake was 600 kcal/day on POD 2 and 1000 kcal/day on POD 3, supplemented with peripheral parenteral nutrition (PPN). In the EN + PN group, EN was started at 200 kcal/day and increased every 2 days to a maximum value of 600 kcal/day. The patients in the EN + PN group received parenteral nutrition by PPN and TPN to compensate for the caloric shortage. Parenteral nuturition was preferentially decreased. Oral intake was started on POD 7. TPN was stopped when the oral intake was over 500 kcal/day, and EN was stopped when the oral intake was over 1000 kcal/day. A unified enteral diet (Isocal; Mead Johnson, Evansville, IN) containing 1000 kcal, 33 g protein, 123 g carbohydrates, and 42 g lipids per liter was administered to the patients. Possible adverse reactions to enteral nutrition were recorded daily. Enteral feeding was reduced or discontinued in case of intolerable emesis, abdominal distention/cramps, or diarrhoea.

### Catheter regimen

A central venous catheter was preoperatively inserted in all the patients; gabexate mesilate 1.5 g/24 h (FOY; Ono, Osaka, Japan) was administered through the catheter for 3 days after the operation. During the operation, all the patients received intra-abdominal drainage and urinary catheter. These catheters were removed as soon as possible, except in 1 case that had serious complications.

### Laboratory and clinical investigations

The operation time, blood-loss volume, and amount of blood transfusion during and after the surgery were carefully recorded. The samples for laboratory investigations were obtained on PODs 1, 3, 5, 7, and 14. The laboratory parameters assessed included the serum levels of total protein, albumin, pre-albumin, and transferrin (as nutritional parameters); lymphocyte counts, T-cell subpopulation (the ratio of CD4 T cells to CD8 T cells, i.e. CD4/CD8), and serum levels of IgG, IgM, and IgA (as immunological parameters); and the serum levels of total bilirubin, cholinesterase, alanine transaminase, aspartate transaminase, lactate dehydrogenase, alkaline phospatase, gamma-glutamyl transpeptidase, amylase, urea nitrogen, and creatinine (as biochemical parameters). Body weight was periodically measured before and after the surgery. Postoperative complications, including surgical-site infection, leakage from anastomose, pancreatic fistula, cholangitis, small-bowel obstruction, delayed gastric emptying (a surgery-related complication), abdominal cramps, distention, diarrhea, and vomiting (an enteral-feeding-related complication) were carefully monitored every day.

### Statistical analysis

The data are expressed as means ± SEM. The statistical significance of the data was determined by unpaired and paired Student's t tests or the chi-square test. P values < 0.05 were considered to be statistically significant. Statistical calculations were performed using Prism Version 4.0 (GraphPad Software Inc.).

## Results

A total of 17 patients were enrolled in this study. The median age of the subjects was 68 years (range, 43–86 years). Eight patients received postoperative nutritional support primarily by enteral nutrition (EN group), and 9 patients received postoperative nutritional support in the form of enteral feeding combined with parenteral nutrition (EN + PN group). Both groups were comparable with respect to patient characteristics, preoperative factors, and preoperative laboratory findings (Table 2). With respect to intraoperative factors, there were no significant differences between the 2 groups in any of the parameters, including operation time, blood loss, number of patients who received blood transfusion, surgical procedure, and histopathological diagnosis (Table 3).

**Table 2:** Preoperative patient characteristics

**Characteristic**	**EN group** **(n = 8)**	**EN+PN group** **(n = 9)**
Age (yr)	66.5 ± 4.8	69.8 ± 3.3
Sex (Male/female)	6/2	6/3
Body weight (kg)	61.3 ± 2.9	55.8 ± 2.2
Body mass index (m^2^)	24.2 ± 1.2	21.9 ± 0.5
Patients with jaundice (%)	25.0	33.3
Patients with diabetes (%)	25.0	11.1
Patients with preoperative PTCD (%)	25.0	22.2
Preoperative laboratory values		
Hemoglobin (g/L)	12.9 ± 0.6	11.7 ± 0.6
Albumin (g/L)	3.6 ± 0.1	3.5 ± 0.1
Bilirubin (mg/dL)	0.9 ± 0.1	0.8 ± 0.1
		
No significant differences noted.		

**Table 3:** Intraoperative factors and histopathology

**Characteristic**	**EN group** **(n = 8)**	**EN+PN group** **(n = 9)**
Duration of surgery (min)	457.4 ± 18.6	564.6 ± 56.6
Operative blood loss (mL)	954.3 ± 155.5	954.4 ± 271.3
Blood transfusion (%)	62.5	77.7
Surgical Procedures (PD/PpPD)	2/6	6/3
Histopathologic finding		
Pancreatic carcinoma	6	7
Cholangio cellular carcinoma	1	1
Chronic pancreatitis	1	1
		
No significant differences noted.		

The overall postoperative morbidity across all the study groups was 53%, and this value did not differ between the 2 groups (Table 1). One patient in the EN + PN group reported with minor pancreatic leakage. Another patient in the EN group reported with minor leakage of the gastrojejunal anastomosis. They conservatively recovered without any treatment. Another patient in the EN group died as a result of acute respiratory distress syndrome caused by infection. There were no differences in other complications, including ileus, anastomotic ulcer, and surgical-site infection, between the 2 groups. The overall mortality was 5.8%.

Although the methods used for postoperative nutrition were different in each group, the total caloric intake in the 2 groups, including the caloric intake attributed to EN+PN, was similar (Fig. 1).

With respect to the factors related to postoperative nutrition, enteral feeding was well tolerated in the patients of the EN + PN group, and few patients exhibited symptoms. In 1 patient (11.1%) in the EN + PN group, enteral feeding was discontinued due to diarrhea before resumption of oral intake. In contrast, in the EN group, enteral feeding was discontinued before resuming oral intake or sufficient oral intake in 5 of the 8 patients (62.5%), primarily due to diarrhea and abdominal distention (Table 4). Furthermore, the duration of enteral feeding in the EN group was significantly shorter than that in the EN + PN group (10.6 ± 2.3 days vs. 23.5 ± 4.4 days, *p *= 0.0255). However, the duration of retention of the central venous line in the EN + PN group was significantly longer than that in the EN group (7.7 ± 1.1 days vs. 12.0 ± 1.5 days, *p *= 0.0418). However, there were no significant differences in the frequency of catheter-related infections between the 2 groups (Table 4). We did not observe any aspiration episodes or enteral-feeding-associated intestinal ischemia. The percentages of weight loss on POD 21 and the lengths of postoperative stay were not different between the 2 groups.

**Table 4:** Factors related postoperative nutrition

	**EN group** **(n = 8)**	**EN+PN group** **(n = 9)**	***P *value**
Nutrition related complications			
Dirrhea	6	4	0.2014
Abdominal distention	7	1	0.0016
Nausea	2	3	0.7066
Removal day of central venous catheter (POD)	7.7 ± 1.1	12.0 ± 1.5	0.0418
Frequency of catheter fever (%)	37.5	22.2	0.4902
Duration of Enteral nutrition (days)	10.6 ± 2.3	23.5 ± 4.4	0.0255
Drop-out rate of EN (%)	62.5	11.1	0.0269
First day of oral intake (POD)	8.8 ± 0.9	8.4 ± 1.2	0.8068
Weght loss ratio on POD 21 (%)	12.9 ± 2.2	12.3 ± 1.7	0.8393
Hospital stay (days)	40.4 ± 5.9	57.2 ± 4.0	0.0826

**Figure 2:**
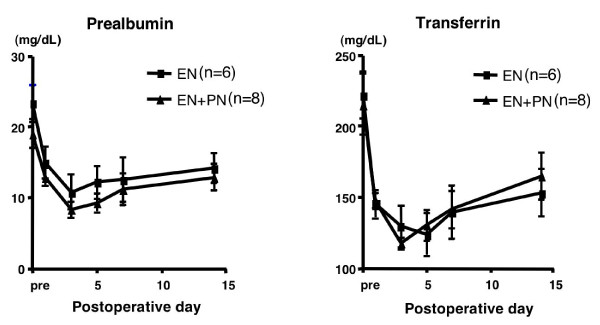
**Comparison of nutritional parameters**. Mean values of prealbumin and transferrin in the EN (square) and EN + PN (triangle) groups. The error bars represent the standard error of the mean (SEM).

**Figure 3:**
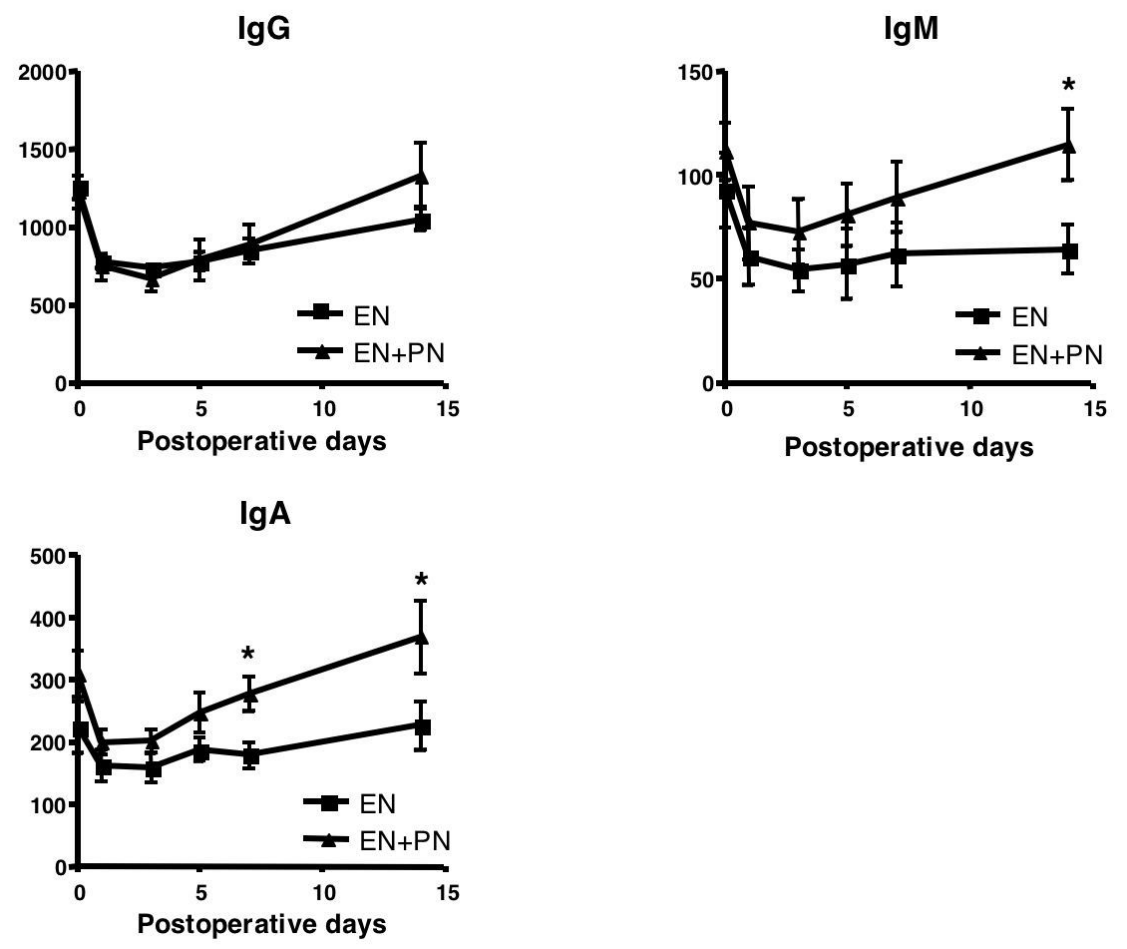
**Comparison of immunological parameters**. Mean values of IgG, IgM, and IgA in the EN (square) and EN + PN (triangle) groups. The error bars represent the SEM; * signifies *p *< 0.05.

**Figure 4:**
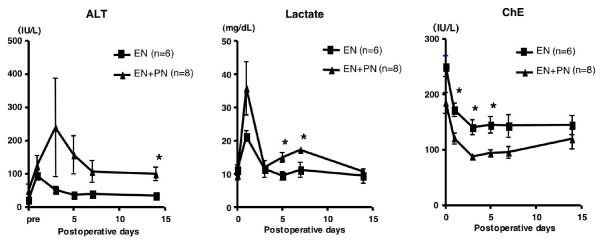
**Comparison of biochemical parameters**. Mean values of ALT, lactate, and ChE in the EN (square) and EN + PN (triangle) groups. The error bars represent the SEM; * signifies *p *< 0.05.

The subanalysis comprised 14 patients (EN group, 6; EN + PN group, 8), after excluding the 2 EN patients and 1 EN + PN patients, in whom enteral feeding could not be continued until POD 5. Among the nutritional parameters, the levels of serum albumin, total protein, albumin, and rapid-turn-over proteins such as pre-albumin and transferrin decreased until POD 3 and increased gradually thereafter, but there was no significant difference between the 2 groups (Fig. 2). Among the immunological parameters, there was no significant difference in the lymphocyte counts and the T-cell subpopulation, i.e. CD4/CD8, between the 2 groups (data not shown). The immunoglobulin levels decreased in the early postoperative days and gradually increased in the late postoperative days, especially in the EN + PN group; at POD 14, there were significant differences between the levels of IgA and IgM in the 2 groups (Fig. 3). Among other biochemical parameters, the levels of ALT were significantly reduced in the EN group on PODs 7 and 14 (Fig. 4). The levels of ChE decreased in the early PODs, especially in the EN + PN group, with significant differences on PODs 1, 3, and 5 (Fig. 4). On PODs 5 and 7, the levels of lactate were significantly low in the EN + PN group (Fig. 4).

## Discussion

PD is associated with a high incidence of postoperative complications even when performed at a high-volume center. While the mortality rate can be reduced to less than 4%, the incidence of postoperative complications continues to range from 35% to 50% in most series. Most patients with pancreatic tumors present with significant weight loss due to anorexia and malabsorption, and they may to undergo a period of inadequate oral intake for up to 10 days after surgery [9,10].

In the last decade, several clinical and experimental studies have reported on the beneficial effects of perioperative enteral nutrition, especially early postoperative enteral feeding, over parenteral and delayed enteral nutrition under critical conditions [1-5]. While the precise mechanisms through which early enteral feeding exerts its positive actions on the outcome are still unclear, the preservation of the integrity of gut structure/function, balanced intestinal microflora, and the maintenance of an effective local and systemic immunocompetence, have been strongly implicated [1-5,8]. Despite these theoretical and clinical advantages, many surgeons remain committed to a postoperative period of "bowel rest", which has long been hypothesized, but never demonstrated to reduce the risk of anastomotic leak. The role of artificial nutrition in affecting morbidity after major pancreatic resection has been markedly neglected. In 1994, Brennan et al. published the first trial on postoperative nutritional support in patients undergoing PD, and they reported that routine postoperative TPN could not be recommended [12]. Since then, many reports have indicated the effect of postoperative enteral nutrition after PD [9,10].

In our unit, which is a high-volume centre of pancreatic surgery, all patients received enteral feeding after surgery. However, there was no guideline for postoperative nutritional support in our unit. Before performing this study, we retrospectively examined the nutritional aspects and postoperateive complaications of 30 patients who underwent PD, including PpPD (not published). In the data obtained from these examinations, the ratio of enteral nutrition drop-outs was high (34.6%), which could be primarily attributed to diarrhea in the early postoperative period, although previous studies reported that approximately 90% of the enterally fed patients reached the full nutritional regimen within 4 days after digestive surgeries, such as esophageal resection and gastrointestinal resection [1-5]. We considered that in PD procedure encompassing the lymph node and/or including ganglion dissection around the celiac artery and the superior mesenteric artery, diarrhoea is a frequent complication. In addition, in our previous studies, there were no significant differences in the nutritional and immunological parameters and the clinical outcomes due to the volume of enteral feeding. Since the volume of enteral feeding was unstable and insufficient, almost all patients tended to undergo prolonged central venous line replacement (median, 14.0 days; range, 5–21 days); consequently, a number of patients showed catheter fever (30.8%). In a recent investigation, it was revealed that the high occurrence of infection-related complications did not result from the route of nutrition (total parenteral nutrition), but was caused by hyperglycemia [6]. Strict blood-glucose control with insulin could lead to the prevention of infectious complications [7]. On the basis of these findings, we considered enteral nutrition combined with parenteral nutrition as a better mode of postoperative nutritional support.

In this clinical pilot study, we primarily aimed to determine the ideal procedure of postoperative nutritional support that would ensure that the patients who underwent PD received sufficient caloric intake without dropping out. In the previous studies, gastrointestinal complications were observed in an unexpectedly high proportion of the patients who received a standard enteral preparation; these complications commonly consisted of nausea, vomiting, abdominal distension, and diarrhea [4,8-10]. Moreover, in several recent postoperative studies on selected patients with esophageal, gastrointestinal, and pancreatic diseases, there has been considerable variation in the results describing the tolerance to enteral feedings [1,3-5,8-10,12]. In these studies, enteral feeding was considered to have been successfully established in more than 70% of the patients. More than 80% of the patients received >600 kcal/day from a standard enteral diet. Almost all that patients achieved a feeding rate of >40 ml/hour. On the basis of these data, we set 600 kcal/day as the maximal dose of enteral feeding in the EN + PN group, and we selected Isocal, which contains sufficient dietary fiber and medium-chain fatty acids, both of which contributed to the reduction in the occurrence of diarrhea as the enteral diet in this study.

We divided the 17 patients into 2 groups according to the mode of postoperative nutritional support: the EN and EN + PN groups. There were no significant differences between the baseline profiles of the 2 groups. Although the routes of administration of the diet were different, the patients of both groups had a similar total caloric intake, and there was no significant difference between the nutritional analysis in the 2 groups. Consistent with these findings, there was no difference between the 2 groups in terms of weight loss on POD 21. The number of patients PD was more in the EN + PN group, while the number of patients who underwent PpPD was more in the EN group. However, we did not consider that this factor would cause any bias in the evaluation of postoperative nutrition, because the jejunal tubes were inserted from the aboral portion of the gastrojejunal anastomosis (i.e. the enteral diet did not pass through the preserved stomach).

In the subanalysis of immunological function, there were no significant differences in the parameters indicating cellular immunity, i.e. leukocytes and lymphocytes counts and the T cell subpopulation. Serum immunoglobulin plays an important role in host humoral immunity. Although the serum levels of IgA and IgM dropped remarkably in all the patients after the operation, they recovered quickly in the EN + PN group and were significantly higher than those in the EN group. However, because of the small number of patients in the present study, it is unclear whether this findings suggests an improvement in the postoperative immunological status. However, there was not inflammation or infection in the patients in the EN + PN group.

In the subanalysis of biochemical parameters, there were no significant differences in any of the parameters, excluding those of hepatic function, between the 2 groups. The postoperative increase in ALT and lactate levels and decrease in the ChE level in the EN +PN group could not be clarified. However, we considered that these changes were caused by TPN, since it has been reported that patients receiving TPN usually show mild-to-moderate elevations in transaminase and alkaline phosphatase levels and hepatic steatosis or portal triaditis on biopsy; the steatosis is reversible, if TPN is administered for a short period [13,14].

Clinically, our most suggestive finding was that more patients of the EN group dropped out of enteral feeding, mainly due to diarrhea and abdominal distention. The patients of the EN + PN group received parenteral nutrition for a longer duraton than the EN group (7.7 ± 1.1 days vs. 12.0 ± 1.5 days, *p *= 0.0418); however, it was also demonstrated that there was no significant difference in the occurrence of catheter-associated infections (3/8 in the EN group vs. 2/9 in the EN + PN group, *p *= 0.4902) under conditions where the central venous route was removed as early as possible.

We suggest that enteral feeding combined with parenteral nutrition may be as safe as total enteral nutrition, which has been reported as the standard method, for ensuring proper completion of postoperative nutrition. Moreover, EN + PN can be a more suitable mode of postoperative nutrition for the patients who have undergone PD. We discontinued our study because many patients in the EN group had to discontinue enteral feeding. However, because of the small number of patients in this study, further studies are required for complete elucidation of these findings.

## Competing interests

The authors declare that they have no competing interests.

## Authors' contributions

SN conceived the study, participated in its design and coordination, and drafted the manuscript. All other author has contributed substantially to the study design and coordinated the study. All authors have read and approved the final manuscript.

## Sources of funds

No external funds. Authors funded this study.
